# Effects of thirty and sixty minutes of moderate-intensity aerobic exercise on postprandial lipemia and inflammation in overweight men: a randomized cross-over study

**DOI:** 10.1186/s12970-016-0137-8

**Published:** 2016-06-29

**Authors:** Sam R. Emerson, Stephanie P. Kurti, Brian S. Snyder, Karthikeyan Sitaraman, Mark D. Haub, Sara K. Rosenkranz

**Affiliations:** Physical Activity and Nutrition Clinical Research Consortium, Department of Food, Nutrition, Dietetics, & Health, Kansas State University, 212 Justin Hall, 1324 Lovers Lane, Manhattan, KS 66506 USA; Department of Kinesiology, 1A Natatorium, Kansas State University, Manhattan, KS 66506 USA

**Keywords:** Cytokines, Triglycerides, High fat meal

## Abstract

**Background:**

The transient rise in blood lipids following a high-fat meal (HFM), known as postprandial lipemia, is linked to systemic inflammation and cardiovascular disease, but can be blunted by exercise. However, minimal research has investigated the effects of realistic exercise bouts on postprandial lipemia and inflammation in at-risk individuals. The purpose of this study was to assess the effects of moderate-intensity aerobic exercise lasting 30 or 60 min performed the evening before a HFM, on postprandial lipemia and inflammation in overweight, insufficiently active men.

**Methods:**

In this randomized-crossover study, twelve participants remained sedentary (CON), or performed a brisk walk on a treadmill at 60 % VO_2peak_ for either 30 min (EX-30) or 60 min (EX-60), after which they consumed a small snack (270 kcal) to partially replace exercise energy expenditure. Following a 12-h overnight fast, participants consumed a standard HFM (1 g fat/kg; 1 g CHO/kg; 1117.8 ± 117.0 kcal). Blood draws were performed at baseline (pre-HFM) and 1, 2, 4, 6, and 8 h post-HFM to assess glucose, insulin, lipids, and systemic inflammation.

**Results:**

There were no significant differences (*p* > 0.05) in fasting triglycerides between EX-60 (118.7 ± 68.3 mg/dL), CON (134.8 ± 66.2 mg/dL) or EX-30 (135.5 ± 85.4 mg/dL). There were no differences in peak, time-to-peak, total or incremental area-under-the-curve between trials for triglyceride response (*p* > 0.05). There was no significant main effect of time (*p* > 0.05) in IL-1ra, IL-4, IL-5, IL-6, IL-10 or TNF-α from baseline to 8 h post-HFM in any trial.

**Conclusions:**

In summary, we found that in overweight, insufficiently active men, neither 30 nor 60 min of moderate-intensity exercise performed 12 h prior to a HFM attenuated postprandial lipemia or inflammation, which could potentially be explained by the partial caloric replacement of exercise energy expenditure.

## Background

A substantial rise in blood lipids following a meal (postprandial lipemia) is associated with cardiovascular disease, visceral adiposity, and insulin resistance [1–3]. Since most individuals spend the majority of the day in a postprandial state, the ability to effectively clear triglycerides following a meal is paramount [4]. Overweight and obese individuals present with increased fasting triglyceride levels [5, 6] and spend more time in the postprandial state [5] compared to normal weight individuals. Further, sedentary populations tend to ingest more dietary fat compared to active counterparts [7]. Since the majority of adults in Western society are overweight or obese [8] and do not meet physical activity guidelines [9], the adverse effects of the persistent and dynamic postprandial state clearly represent widespread metabolic dysfunction.

Similar to postprandial lipemia, chronic inflammation is recognized as a major risk factor in the development of cardiovascular disease [10] and the metabolic syndrome [11]. Pro-inflammatory cytokines are known to increase during the postprandial state [12], as well as to be chronically elevated in individuals who are overweight or obese [13]. Thus, an interrelationship exists between lipid metabolism, cardiovascular disease, body composition, and the immune system. However, research has rarely examined the postprandial lipemic response in conjunction with the inflammatory response in “at-risk” populations.

Acute exercise routinely decreases postprandial lipemia in apparently healthy, normal weight individuals [4]. In studies utilizing exercise protocols of extensive duration and energy expenditure (e.g. 90 min to expend ~700–900 kcals) [14] the evening before a high-fat meal, there is almost a universal lowering of postprandial triglycerides [15]. Further, studies implementing exercise protocols of longer duration (e.g. 3 h) [16, 17] have demonstrated anti-inflammatory effects of exercise as well. However, the aforementioned exercise sessions are often unfeasible or undesirable for the majority of the population (i.e. overweight and obese individuals) to perform on a daily basis, making the applicability of these findings questionable. The American College of Sports Medicine (ACSM) recommends at least 150 min of moderate-intensity exercise per week, which can be met through 30–60 min of moderate-intensity exercise five days per week [18]. Thus, there is currently a disconnect between research investigating the beneficial effects of acute exercise and feasible expectations for at-risk individuals to realize these benefits. However, there are data suggesting that individuals of lower aerobic capacity may require less energy expenditure during exercise in order to blunt postprandial lipemia [19]. Further, there have been several studies demonstrating blunted postprandial lipemia following 60 min of aerobic exercise [20–22]. Thus, although many studies have utilized protocols that may not be realistic for individuals in daily life, there is evidence to suggest that modest amounts of exercise may substantially attenuate postprandial lipemia. To our knowledge, no current studies have investigated the potential lipid-lowering effects of realistically-performed aerobic exercise on both postprandial lipemia and inflammation.

The purpose of this study was to investigate the effects of realistic acute aerobic exercise (aligning with ACSM physical activity guidelines) [18] on postprandial lipemia and inflammation in overweight individuals. We also aimed to compare two exercise sessions of similar intensity but differing durations (30 min versus 60 min) and energy expenditures, to determine whether there was a dose-response relationship between exercise and postprandial lipemia and inflammation in overweight individuals. We hypothesized that: 1) 60 min of moderate-intensity walking would be sufficient to blunt the postprandial lipemic response to a high-fat meal compared to control; 2) 30 min of brisk walking would be insufficient to blunt the postprandial lipemic response; and 3) there would be a correlation between the postprandial lipemic and systemic inflammatory responses to the high-fat meal.

## Methods

### Participants

Twelve healthy, non-smoking, overweight, insufficiently active men (age: 21–35 years) participated in this study. Inclusion criteria for the recruitment and enrollment of participants were: 1) a BMI ≥ 25 kg/m^2^; 2) fasting triglycerides < 150 mg/dL; 3) exercising < 2 days per week with no more than 1 h of moderate- to vigorous-intensity exercise per week; 4) non-smokers (for at least the past year); and 5) not diagnosed with cardiovascular disease. Participants were excluded if they presented with impaired glucose tolerance (see below). All study procedures, including a written informed consent, were reviewed and approved by the Institutional Review Board of the Kansas State University.

### Initial assessment

To determine study eligibility, 5 to 7 days prior to their first trial, participants underwent an oral glucose tolerance test (OGTT) by consuming a 75 g glucose drink after at least an 8 h fast. Blood glucose was measured fasting and 2-h post glucose consumption in order to exclude participants presenting impaired glucose tolerance, impaired fasting glucose, or frank diabetes. Participants then underwent a Dual Energy X-ray Absorptiometry (DEXA) scan (Prodigy software version 5.6, GE Lunar, Milwaukee, WI) to determine body composition. The participants also performed an incremental exercise test to volitional fatigue to determine peak oxygen uptake (VO_2peak_) using the modified Bruce protocol. A metabolic cart (Truemax 2400, Parvo-medics, Provo, UT) was used to monitor expired gases via breath-by-breath analysis. Metabolic and ventilatory data were pooled in 30-s intervals and averaged. The highest VO_2_ attained during the test was considered the VO_2peak_. Heart rate data were gathered during the test using a wireless telemetry system (Polar USA, Woodbury, NY). The incremental test was used to determine exercise intensities in study exercise trials (see below). At the conclusion of the initial assessment, participants received a standardized quantity of food (ready-to-eat frozen meals) based on estimated individual energy needs for the 2 days prior to their first trial.

### Study design

**Fig. 1:**
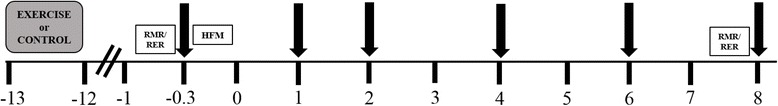
Schematic of study protocol. Participants either exercised (EX-30, EX-60) or remained sedentary (CON) the evening before (~12 h) the meal tolerance test. Resting metabolic rate (RMR) and respiratory exchange ratio (RER) were assessed for 30 min upon arrival at the laboratory the next morning, and again between hours 7 and 8 post-high fat meal (HFM). Blood draws (black arrows) were performed at baseline and 1, 2, 4, 6, and 8 h after the HFM. The HFM was consumed immediate after the baseline blood draw. RMR, resting metabolic rate; RER, respiratory exchange ratio; HFM, high-fat meal

A randomized cross-over design was used in the present study. Following the initial assessment, participants completed three randomized trials separated by at least 7 days. All food was provided to the participants for 2 days prior to every meal tolerance test in order to control caloric and macronutrient intake. The Harris-Benedict equation (activity factor = 1.5) was used to calculate the daily calorie requirement and ensure that participants were in approximate energy balance at the beginning of each trial. Prior to the second and third trials, participants consumed the same foods that were consumed before the first trial. The trials included two exercise trials (brisk walk on a treadmill at 60 % VO_2peak_ for 30 and 60 min; EX-30 and EX-60, respectively) and a control trial (60 min of passive activity; CON). Following EX-30, participants rested passively for the remaining 30 min; thus, all trials were 60 min in duration. EX-60, EX-30, and CON were each completed in the evening. The standardized high-fat meal (HFM) was performed the next morning, approximately 12 h following the exercise condition. Participants observed an overnight fast throughout this period. A visual representation of the study design is displayed in Fig. 1.

### Exercise trials

During the EX-30 and EX-60 trials, the participants walked briskly on a treadmill at approximately 60 % of their previously determined VO_2peak_. Exercise durations of 30 and 60 min (EX-30 and EX-60, respectively) were chosen to reflect physical activity guidelines [18]. This exercise intensity was chosen because it is in the higher range of recommended intensities for individuals of lower cardiorespiratory fitness and a higher intensity appears to be needed to produce the energy expenditure necessary for a reduction in post-prandial lipemia [23]. The heart rate corresponding to 60 % VO_2peak_ was fixed as the target heart rate. Heart rate was continuously monitored throughout the exercise trial and the treadmill speed and incline were adjusted as necessary to match the target heart rate. Also, gas exchange analysis was obtained at specific intervals (minutes 26–30 at each time frame) during the exercise intervention in order to verify the exercise intensity and account for heart rate drift via adjustment of treadmill speed/gradient. Participants then consumed their final meal of the day (Little Debbie Swiss Rolls; 270 kcal, 12 g fat) in the laboratory to ensure a standardized 10-h overnight fast before the HFM the next morning; this evening snack was consumed in all trials (EX-60, EX-30, and CON).

### Meal tolerance test

Figure 1 displays the protocol for the CON, EX-30, and EX-60 trial days. Each participant arrived in the laboratory at ~0600 on the morning of the HFM. Resting metabolic rate (RMR) and respiratory exchange ratio (RER) were measured via indirect calorimetry (Truemax 2400, Parvo-medics, Provo, UT) for 30 min at baseline in the fasted state as well as 460 min after ingestion of the HFM. The measurement of REE and RER were used to assess differences in energy expenditure and substrate oxidation between trials. Participants then consumed the HFM, which consisted of an ice cream plus whipping cream meal composed of 1 g/kg body weight of carbohydrates and 1 g/kg body weight of fat. The caloric content of the test meal was 1117.8 ± 117.0 kcal. Participants consumed 300 mL of fluid with the meal, and did not have difficulty completing the HFM. The participants were sedentary during the HFM period. RMR and RER analyses were performed for 30 min 8-h post HFM.

### Blood samples

Blood samples (10 mL) were collected at baseline (immediately prior to the HFM) and serially 1, 2, 4, 6 and 8 h post-HFM. Samples were collected from an indwelling catheter in the forearm vein into evacuated tubes (10 mL) containing ETDA. The catheter was kept patent by a slow flow of normal saline. The samples were centrifuged and the plasma was frozen immediately with liquid nitrogen and kept at −70 °C. Blood samples were analyzed for triglycerides, glucose, insulin, and inflammatory markers (IL-1ra, IL-4, IL-5, IL-6, IL-10, and TNF-α). Triglycerides were analyzed using spectrophotometry (kit #343-25P, Sigma Diagnostics, St. Louis, MO). Assessment of triglycerides via spectrophotometry has been reported to be accurate within ±5 % [24]. Glucose was analyzed via the glucose oxidase method using an automated analyzer (YSI 2300, Yellow Springs Instruments, Yellow Springs, OH). Insulin was analyzed using a commercially available EIA kit (ALPCO Diagnostics, Windham, NH). This specific commercial EIA insulin kit reported a CoV of ~5.1 %. Commercially available multiplex cytokine assay kits featuring premixed magnetic beads panels were used to analyze inflammatory biomarkers (HT17MG-14 K-PX25, EMD Millipore, Billerica, MA). The principle of the bead-based multiplex assay is as follows: color-coded beads that are coated with a capture antibody specific to each certain analyte are added to the plasma and the analyte-specific antibodies on the beads link to the desired analyte. Next, biotinylated analyte-specific detection antibodies are added to the plasma/solution, forming an antibody-analyte-antibody sandwich. Phycoerythrin-conjugated Streptavidin is added to the plasma. The beads are then read via a commercial multiplex analysis platform (Luminex IS100, Luminex, Austin, TX), which utilizes a dual-laser system. One laser determines the analyte that is being detected on a given bead, while the other laser measures the magnitude of the phycoerythrin signal, which is proportional to the amount of bound analyte. All samples were run in duplicate on the same plate. The intra- and inter-assay CoV for cytokine assessment utilizing this methodology is typically <10 % and <15 %, respectively [25]. The lower limit of quality (LLOQ) varies by cytokine, but is generally < 3 pg/mL [26]. Two IL-4 samples were below the strict LLOQ for IL-4 (3.2 pg/mL), but were above the relaxed LLOQ (0.2 pg/mL), and were thus retained in the analyses [26]. No other cytokine data points were lower than their respective LLOQ [26].

### Statistical analyses

Calculations and analyses were performed using SPSS Statistics software (v.22; SPSS, Inc; IBM Corporation; Armonk, NY). Generation of figures and certain statistical analyses were performed using Graphpad Prism (Version 6.05; GraphPad Software, Inc; La Jolla, CA). A two-way (trial by time) ANOVA with Tukey post-hoc pairwise comparisons was utilized to test temporal and trial differences in REE and RER. AUC was determined via the trapezium rule using GraphPad Prism. Briefly, AUC via the trapezium rule represents the area under the concentration versus time curve divided by the length of the postprandial period, which in this case is 8 h. The area is estimated by splitting the area beneath the curve into several trapeziums of which the areas can be calculated, and summing all of them together. Total AUC represents the total area between y = 0 and the concentration versus time curve, whereas incremental AUC represents the time-averaged postprandial concentration minus the fasting concentration. A one-way (trial) repeated measures ANOVA was used to determine main effects between the three trials (CON, EX-30, EX-60) with regard to peak, time to peak, and AUC data. A two-way (trial by time) repeated measures ANOVA with Tukey’s post-hoc pairwise analysis was used to test differences between trials at each time point during the postprandial period. Cytokines were analyzed using one-way repeated measures ANOVA (time as the within-participants factor). If main effects were found, post-hoc Bonferroni testing was used to test pairwise comparisons. Pearson product moment correlation coefficient for parametric, or Spearman rho for nonparametric data, was used to test relationships between triglycerides and cytokines over time. When, assessing the relationship between triglyceride and cytokine changes, Bonferroni corrections were used to adjust the alpha level when multiple comparisons were made. Data in tables are presented as Mean ± SD, while data in figures are presented as Mean ± SE. Statistical significance was accepted at a type 1 error rate of 5 %.

## Results

### Participant characteristics

Table 1 presents participant characteristics, including age, height, weight, BMI and body fat percentage. Of the 12 participants studied, ten were overweight and two were obese. In the EX-30 trial, participants expended 291.3 ± 70.9 kcal during exercise. In the EX-60 trial, participants expended 582.5 ± 141.8 kcal during exercise.

**Table 1:** Participant characteristics

	Mean ± SD	Range
Age (years)	24.5 ± 5.1	21–35
Height (m)	1.76 ± 0.07	1.66–1.87
Weight (kg)	86.0 ± 9.0	73.9–97.4
Body mass index (kg/m^2^)	27.6 ± 2.3	25.0–31.0
Body fat (%)	32.3 ± 5.9	24.4–39.3
Trunk fat (%)	36.5 ± 6.0	28.0–43.3
VO_2peak_ (mL/kg/min)	38.9 ± 7.9	27.2–51.5

*VO*_*2peak*_ peak aerobic capacity

### Energy balance

Data for energy expenditure and substrate utilization are displayed in Table 2. REE and RER were measured twice during every HFM trial: prior to the HFM and approximately 8 h after the HFM. REE at baseline was significantly lower than 8 h post-HFM for CON (*p* = 0.006 and EX-30 (*p* = 0.02) but not EX-60 (*p* = 0.08). Similarly, RER was significantly higher at baseline compared to 8 h post-HFM in CON (*p* = 0.002) and EX-30 (*p* = 0.006), while there was no difference between baseline and 8 h post-HFM in EX-60 (*p* = 0.32). There were no between-trial differences with regard to REE (*p* = 0.86) and RER (*p* = 0.76) at either time point.

**Table 2:** Energy expenditure and substrate utilization

	CON	EX-30	EX-60	*p*-value
REE (kcal/day)				
Baseline	1573.9 ± 271.8	1619.0 ± 176.8	1620.4 ± 188.2	0.0008
8-h Post-HFM	1817.1 ± 208.0*	1808.7 ± 254.0*	1766.4 ± 265.0	
RER				
Baseline	0.86 ± 0.05	0.86 ± 0.07	0.83 ± 0.07	0.0051
8-h Post-HFM	0.80 ± 0.06*	0.80 ± 0.07*	0.80 ± 0.08	

Resting energy expenditure (REE) and respiratory exchange ratio (RER) were assessed for 30 min at baseline and approximately 8 h post-HFM in each trial. Data are presented as Mean ± SD. P-value column represents change (time effect) from baseline to 8-h after the high- fat meal for the given variable (assessed via two-way ANOVA). *Significantly different from baseline (*p* < 0.05). There were no between-trial differences for either REE (*p* = 0.86) or RER (*p* = 0.76) at any time point. See Results section for more details

*REE* resting energy expenditure; *RER* respiratory exchange ratio

### Fasting metabolic values

Fasting values for triglycerides, insulin, and glucose are displayed in Table 3. All individual values for CON were normal with regard to fasting insulin (<1010 pg/mL), while two participants had abnormal fasting glucose (>100 mg/dL) and two participants presented with abnormal fasting triglycerides (>150 mg/dL). There were no significant differences between any trials for fasting triglycerides (*p* = 0.15), insulin (*p* = 0.47), or glucose (*p* = 0.15).

**Table 3:** Postprandial metabolic data

	CON	EX-30	EX-60	*p*-value
Triglycerides				
Fasting (mg/dL)	134.8 ± 66.2	135.5 ± 85.4	118.7 ± 68.3	0.15
Peak (mg/dL)	302.0 ± 162.7	308.1 ± 173.6	233.9 ± 124.7	0.11
Time to peak (hours)	4.9 ± 1.4	4.7 ± 1.3	3.5 ± 2.3	0.10
AUC-tot (mg/dL × 8 h)	1870.3 ± 869.9	1818.5 ± 1037.3	1444.4 ± 800.6	0.08
AUC-inc (mg/dL × 8 h)	721.0 ± 494.0	737.6 ± 478.3	516.6 ± 303.9	0.24
Insulin				
Fasting (pg/mL)	92.6 ± 37.6	80.6 ± 38.9	81.5 ± 30.4	0.47
Peak (pg/mL)	351.3 ± 128.3	344.8 ± 149.1	317.3 ± 113.8	0.41
Time to peak (hours)	1.2 ± 0.4	1.3 ± 0.5	1.2 ± 0.4	0.26
AUC-tot (pg/mL × 8 h)	1434.2 ± 563.0	1330.4 ± 510.5	1434.9 ± 500.5	0.29
AUC-inc (pg/mL × 8 h)	696.3 ± 467.0	685.9 ± 374.6	783.9 ± 438.8	0.63
Glucose				
Fasting (mmol/L)	5.21 ± 0.32	5.03 ± 0.32	4.86 ± 0.46	0.15
Peak (mmol/L)	5.98 ± 0.74	6.15 ± 1.04	6.33 ± 1.13	0.74
Time to peak (hours)	1.1 ± 0.7	2.1 ± 1.6	3.0 ± 1.2^*^	0.001^a^
AUC-tot (mmol/L × 8 h)	40.43 ± 2.29	41.15 ± 3.50	41.31 ± 3.01	0.79
AUC-inc (mmol/L × 8 h)	−1.26 ± 2.55	1.22 ± 3.29	1.86 ± 5.32	0.18

The above table displays the fasting and postprandial data for triglycerides, insulin, and glucose for the CON, EX-30, and EX-60 trials. Data are presented as Mean ± SD

*AUC-tot* total area under the curve; *AUC-inc* incremental area under the curve

^*^ Significantly different from CON (*p* < 0.05)

^a^ Significant trial effect (one-way ANOVA)

### Postprandial metabolic responses

**Fig. 2:**
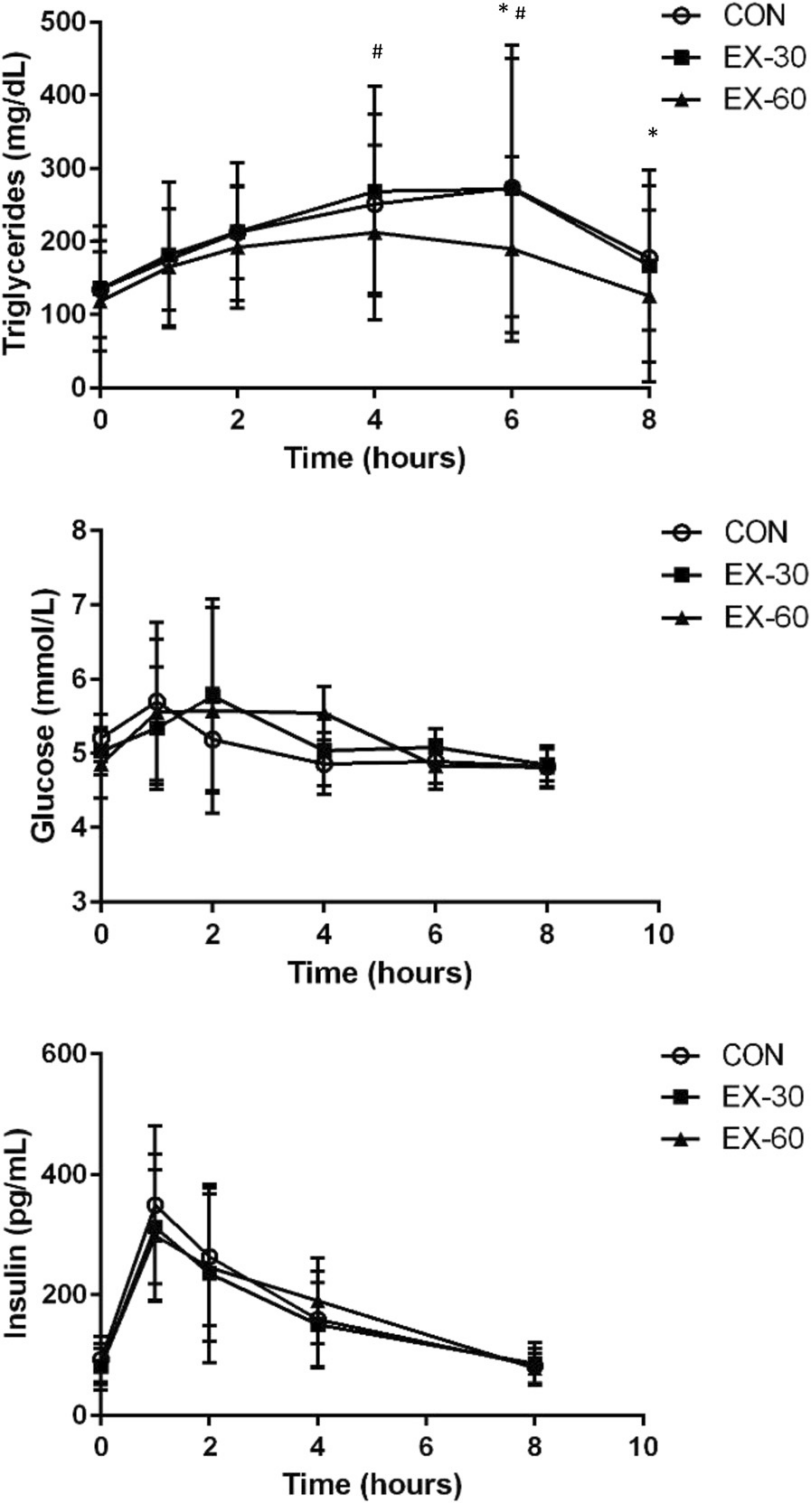
Hourly metabolic responses to the high-fat meal. These figures display the hourly triglyceride (top panel), glucose (middle panel), and insulin (bottom panel) responses to a high-fat meal in the CON, EX-30, and EX-60 trials. Blood draws were performed at baseline (time 0) and serially 1, 2, 4, 6, and 8 h after the high fat meal. Data are presented as Mean ± SE. In two-way repeated measures ANOVA, there were no significant trial effects for triglycerides, glucose, or insulin (*p* > 0.05), although there were some between-trial differences with regard to triglycerides at certain time points. * CON significantly different than EX-60. ^#^ EX-30 significantly different than EX-60

Table 3 also displays peak, time to peak, total area under the curve (AUC-tot), and incremental area under the curve (AUC-inc) responses to the CON, EX-30, and EX-60 trials for triglycerides, insulin, and glucose. Pairwise comparisons indicated that there were no differences with regard to peak (CON vs EX-30, *p* = 0.97; CON vs EX-60, *p* = 0.29; EX-30 vs EX-60, *p* = 0.14), time to peak (CON vs EX-30, *p* = 0.90; CON vs EX-60, *p* = 0.19; EX-30 vs EX-60, *p* = 0.23), AUC-tot (CON vs EX-30, *p* = 0.96; CON vs EX-60, *p* = 0.16; EX-30 vs EX-60, *p* = 0.17), or AUC-inc (CON vs EX-30, *p* = 0.98; CON vs EX-60, *p* = 0.49; EX-30 vs EX-60, *p* = 0.32) between trials for triglyceride responses. Figure 2 (top panel) shows the triglyceride response versus time curve for the three trials. Qualitatively, the triglyceride curves for CON and EX-30 were nearly identical. In comparison, the EX-60 triglyceride curve appears to have peaked earlier and demonstrates an overall blunted response, although not significantly different (*p* > 0.05) from the other two trials.

There were no significant differences between trials with regard to insulin peak, time to peak, AUC-tot, or AUC-inc (Table 3). The insulin response versus time curve for the three trials (Fig. 2, bottom panel) shows qualitatively similar responses for CON, EX-30, and EX-60, with insulin curves for the three trials peaking at 1-h post-meal and then continually decreasing.

Peak glucose response was not significantly different between trials (Table 3). However, there was a significant trial effect for glucose time to peak (*p* = 0.001). In post-hoc pairwise comparisons, time to peak was significantly greater (*p* = 0.007) in EX-60 compared to CON. There were no differences in glucose time to peak between CON and EX-30 (*p* = 0.33) or EX-30 and EX-60 (*p* = 0.35). There were no significant differences for glucose AUC-tot or AUC-inc between trials.

### Markers of inflammation

**Fig. 3:**
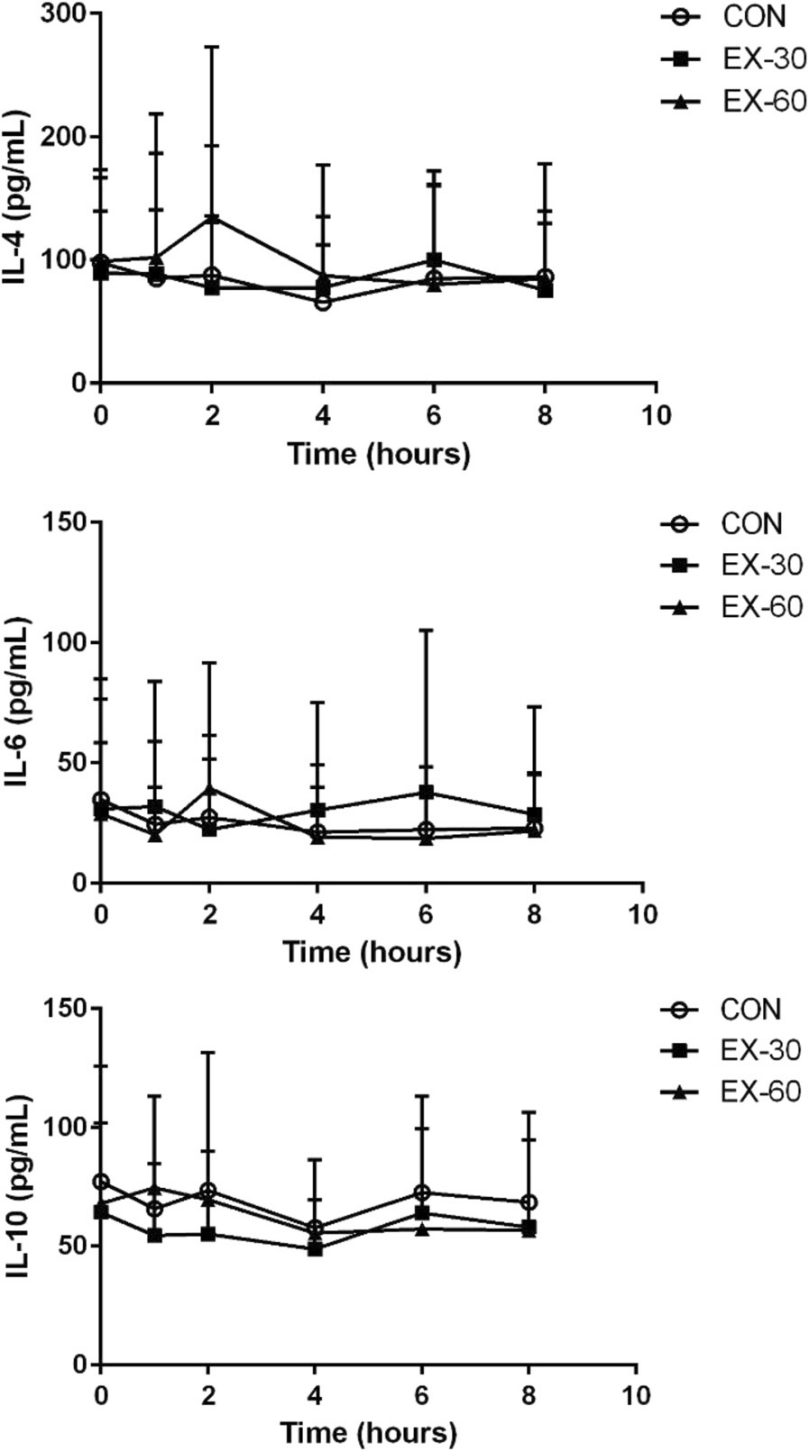
Hourly inflammatory responses to the high-fat meal. These figures display the hourly interleukin (IL)-4 (top panel), IL-6 (middle panel), and IL-10 (bottom panel) responses to a high-fat meal in the CON, EX-30, and EX-60 trials. IL-4, IL-6, and IL-10 are commonly assessed cytokines in previous studies. Blood draws were performed at baseline (time 0) and serially 1, 2, 4, 6, and 8 h after the high fat meal. Error bars reflect standard error (SE). There were no significant trial effects for any cytokines (*p* > 0.05), nor were there trial differences at any time points. IL, interleukin

Figure 3 shows hourly changes following HFM consumption for three of the more commonly assessed cytokines (IL-6, IL-4, and IL-10) in each trial. Table 4 displays fasting, peak, and time to peak values for IL-1ra, IL-4, IL-5, IL-6, IL-10, and TNF-α in the CON, EX-30, and EX-60 trials. There were no significant changes in any of the assessed inflammatory markers post-HFM in CON, EX-30 or EX-60 as a main-effect of time (baseline to 8 h post-HFM) or between conditions.

**Table 4:** Postprandial inflammatory data

	CON	EX-30	EX-60	*p*-value
IL-1ra				
Fasting (pg/mL)	193.8 ± 135.4	202.2 ± 117.7	186.4 ± 98.5	0.95
Peak (pg/mL)	260.8 ± 183.2	300.0 ± 212.6	375.5 ± 359.9	0.26
Time to peak (hours)	4.1 ± 3.5	3.8 ± 3.0	2.3 ± 2.6	0.34
IL-4				
Fasting (pg/mL)	97.9 ± 75.5	89.3 ± 50.3	98.8 ± 68.0	0.63
Peak (pg/mL)	126.2 ± 95.9	118.2 ± 66.4	158.3 ± 132.7	0.65
Time to peak (hours)	3.0 ± 3.4	2.5 ± 2.5	2.1 ± 2.6	0.23
IL-5				
Fasting (pg/mL)	2.6 ± 1.0	2.5 ± 0.7	2.8 ± 0.8	0.72
Peak (pg/mL)	2.9 ± 1.0	2.8 ± 0.8	3.2 ± 1.1	0.23
Time to peak (hours)	1.8 ± 2.0	3.9 ± 3.1	3.6 ± 3.5	0.18
IL-6				
Fasting (pg/mL)	34.7 ± 50.4	30.8 ± 45.8	28.8 ± 29.7	0.92
Peak (pg/mL)	35.5 ± 47.9	47.7 ± 68.7	44.1 ± 50.3	0.73
Time to peak (hours)	1.6 ± 2.8	2.5 ± 3.0	1.8 ± 2.7	0.63
IL-10				
Fasting (pg/mL)	77.1 ± 48.5	64.2 ± 37.7	67.9 ± 35.1	0.30
Peak (pg/mL)	98.4 ± 48.3	79.6 ± 36.9	96.7 ± 57.0	0.23
Time to peak (hours)	2.7 ± 3.1	4.2 ± 3.1	2.8 ± 2.9	0.50
TNF-α				
Fasting (pg/mL)	9.1 ± 6.6	10.1 ± 8.9	9.7 ± 5.7	0.47
Peak (pg/mL)	10.9 ± 6.4	12.8 ± 12.6	12.4 ± 8.2	0.72
Time to peak (hours)	2.7 ± 3.0	2.3 ± 2.6	1.8 ± 2.6	0.49

The above table displays the fasting, peak, and time to peak data for interleukin (IL)-1ra, IL-4, IL-5, IL-6, IL-10, and TNF-α for the CON, EX-30, and EX-60 trials. Data are presented as Mean ± SD

No significant trial effects

*IL* interleukin

We also assessed associations between changes in cytokines and changes in triglycerides to determine differences in inflammatory responses between conditions. Bonferroni corrections were used when making multiple comparisons between time and condition, and the alpha level was adjusted accordingly. For anti-inflammatory cytokines, the alpha level was set at *p* < 0.006 and for pro-inflammatory cytokines the alpha level was set at *p* < 0.004. The only statistically significant change following Bonferroni correction was in IL-6, which was associated with the change in triglycerides in the EX-30 condition (*r* = 0.820, *p* = 0.002). There were no significant associations between changes in cytokines and triglycerides in any of the other cytokines between conditions after Bonferroni correction.

## Discussion

### Main findings

The primary finding of the current study was that, contrary to our hypothesis, 60 min of moderate-intensity aerobic exercise did not result in attenuation of the postprandial lipemic response when performed 12 h prior to a HFM. Thirty minutes of exercise, somewhat reflective of current physical activity guidelines (>150 min of moderate-intensity exercise per week [18]), was ineffective at reducing both fasting triglycerides and also postprandial lipemia in response to a HFM. Interestingly, there were no significant differences between trials with regard to postprandial inflammatory cytokines. Further, the ability to test the relationship between postprandial lipemia and inflammation may have been weakened by the minimal between-trial differences in triglycerides.

### Effects of exercise on postprandial lipemia

It is well-established that consumption of a fatty meal can result in a substantial rise in blood lipids, and that this lipemia has a basis in atherosclerosis [1, 27]. It is also widely acknowledged that acute aerobic exercise performed 10–12 h prior to a high-fat meal is typically effective in attenuating postprandial lipemia [15]. In this context, over the past few decades, research has been dedicated to better understanding the specific relationship between acute pre-meal exercise and postprandial lipemia via modification of variables such as exercise duration, intensity, mode, and timing, as well as meal and subject variables [15]. To our knowledge, the present investigation is the first to specifically test the effectiveness of an acute bout of exercise reflective of current physical activity recommendations [18] on postprandial lipemia and inflammation in a group of at-risk individuals (i.e. overweight men). We found that the historically recommended 30 min of aerobic exercise, when performed 12 h prior to a high-fat meal, was ineffective in blunting both fasting triglycerides and postprandial lipemia in overweight men. This finding is in agreement with the findings of Pfeiffer et al. [28] who also found that 30 min of walking at 50 % VO_2max_ was ineffective in blunting postprandial lipemia in healthy young men that performed cycling exercise immediately prior to the ingestion of two mixed test meals. On the other hand, several studies have shown a beneficial effect of 30 min of aerobic exercise on postprandial lipemia [29, 30]. However, differences in protocols are the likely explanations for the disagreement in findings, considering that Klein et al. [30] used a higher-intensity exercise (75 % VO_2max_) performed after the high-fat meal and Miyashita and Tokuyama [29] used a moderate-fat meal in healthy participants (versus a high-fat meal in overweight participants, as in the present study). Similarly, one study found that an exercise bout producing an energy expenditure of less than 500 kcal significantly blunted postprandial lipemia, although the exercise bout was 90 min in duration and the participants had low aerobic capacities (VO_2max_ ~ 30 ml/kg/min) [19].

However, the more surprising finding in our study was that 60 min of exercise was not effective for blunting postprandial lipemia (AUC-tot or AUC-inc). This null finding disagrees with several studies that have reported a postprandial lipid-lowering effect of 60 min of exercise [20–22]. There are a noteworthy number of investigations in addition to the present study that have not found reduced postprandial lipemia after an hour of exercise [28, 31–34]. Clearly, the lipid-lowering effect of 60 min of exercise is not universal, nor consistently confirmed in the literature. These discrepancies are certainly affected by such study design variables as meal timing, caloric content, and composition; exercise duration, intensity, and energy expenditure, as well as participant factors such as fitness and health status. However, while the present study did not find a significantly blunted lipemic response in the EX-60 condition relative to the other conditions, there were qualitative improvements in postprandial lipemia following 60 min of exercise (Fig. 2). Specifically, there was a “moderate effect” in the EX-60 condition, with the effect size (Cohen’s *d*) for CON vs EX-60 in terms of AUC-tot and AUC-inc lipemic responses being 0.51 and 0.50, respectively.

Energy balance can play a critical role in determining whether exercise blunts postprandial lipemia [35–37]. In the present study, participants consumed a small snack of 270 kcal after the exercise session/before leaving the laboratory. Thus, in EX-30 the energy deficit (291.3 ± 70.9 kcal) would have been nearly completely replaced, and in EX-60 the energy deficit (582.5 ± 141.8 kcal) would have been partially replaced. This replacement of energy deficit may explain the lack of effect of exercise on postprandial lipemia and the contradictory findings between the present study and previous studies that found a lipid-lowering effect of 60 min of exercise [20–22].

There are also other study design features that may explain why previous investigations found that 60 min of exercise lowered postprandial lipemia and the current investigation did not. For example, Dekker et al. [22] studied obese, hypertriacylglycerolemic men, who would display an elevated, prolonged postprandial lipemia and thus a more pronounced effect for exercise. Kolifa et al. [21] had participants cycle at 70–75 % of maximal heart rate, leading to a greater exercise energy expenditure, and Thomas et al. [20] used a test meal of 1.5 g fat/kg (150 % fat content of present study). We believe that our protocol is consistent with the majority of postprandial lipemia investigations [15], reflective of realistic meal consumption and exercise intensity and duration for overweight individuals, and in line with current physical activity guidelines [18]. In light of this, our findings that 30 and 60 min of moderate-intensity aerobic exercise were ineffective in attenuating postprandial lipemia have important public health implications, and could be considered a caveat to our traditional physical activity recommendations for adults. It appears that exercise 12 h prior to a high-fat meal, even when expending more than 500 kcal, may be insufficient for attenuating postprandial lipemia if calories are completely or partially replaced following exercise. Less fit individuals may indeed require less exercise energy expenditure to blunt postprandial lipemia [19], but this may need to be in the context of non-replacement of the exercise-induced energy deficit; otherwise a greater exercise energy expenditure may be required.

### Circulating markers of inflammation

Previous studies have suggested that inflammatory cytokines increase after a HFM in overweight participants [38], however this is not a consistent finding due to variability in many studies, as well as differential time course for measurements [39]. In the current study, we assessed cytokines from 10 min prior to a HFM (1 g/kg fat and 1 g/kg CHO) to 8 h post-HFM. With the large time span from baseline to 8 h after the high-fat meal, it was unexpected to find an increase in PPL without significant changes in the cytokines measured. However, the inflammatory cytokine literature is conflicting and several factors may explain the increase in PPL without a change in pro-inflammatory cytokines. Specifically, although the rise postprandial triglycerides has been reported to induce inflammation [40], this relationship between changes in triglycerides may not always be associated with changes in inflammation [41–43].

Although a 60-min moderate bout of exercise has been reported to attenuate PPL as previously stated, an acute bout of physical activity may still have anti-inflammatory effects without attenuating PPL. These anti-inflammatory actions may occur by increasing IL-6 from the contracting muscle [13]. Release of IL-6 may promote an anti-inflammatory environment by increasing downstream release of anti-inflammatory cytokines such as IL-10, and attenuating subsequent increases in TNF-α [13]. Although changes in cytokines in the current study were not significantly different between trials and there was large inter-subject variability, peak anti-inflammatory IL-4 displayed marginal differences in EX-60 compared to EX-30 and CON (IL-4 95 % CIs; EX-60: 39.895–166.904 pg/mL: EX-30: 48.988–120.508 pg/mL: CON: 36.336–132.860 pg/mL). Therefore, although there were no statistically significant changes over time, exercise may have increased the anti-inflammatory environment, thereby increasing IL-4.

It is well established that the generation of reactive oxygen species and subsequent increases in circulating inflammatory cytokines occur following consumption of a HFM [44, 45] and also following acute exercise [46] in overweight individuals compared with lean controls. Specifically, Aljada and colleagues [44] reported that IL-6 increased from baseline to 2 h post-HFM in lean and obese individuals; however, IL-6 remained elevated in obese individuals only. In Fig. 3 from our data, it appears the peak IL-6 occurred similarly to the lean participants described in Aljada and colleagues’ previous work [44], whereas the peak IL-6 response in EX-30 qualitatively increased more slowly and remained elevated until 6 h post-HFM. These considerations may also help to elucidate why IL-6 was only associated with a change in triglycerides in the EX-30 condition. Although IL-6 may act as an anti-inflammatory cytokine post-exercise [13], it can also act as a pro-inflammatory cytokine post-HFM [47]. Our laboratory has reported that exercise, either 12 h before a HFM or 30 min after a HFM, may not attenuate postprandial lipemia [48–50]. In both studies, there were no reported changes in cytokines post-HFM. In the current study, the finding that IL-6 did not increase post-HFM in CON was surprising, although it was alignment with recent studies [39] that showed a decrease in IL-6 from baseline to 2 h following a high-fat meal before becoming elevated above baseline. Therefore it appears that many factors influence the actions of these cytokines, and further investigations should be performed to elucidate the time course of inflammation (both in terms for pro- and anti-inflammatory cytokines), as well as interactions between cytokines when investigating postprandial inflammation.

### Strengths and experimental considerations

The present study is novel in that it employs exercise bouts that may be reasonable for individuals in the general population to complete. As stated previously, the majority of previous postprandial lipemia and inflammation investigations have used exercise protocols that may not be realistic for many individuals to take part in on a regular basis. Also, the current study included at-risk (overweight or obese) individuals, a relevant study population. Other strengths of the present study are the 8-h measurement duration in the postprandial period, and the large number of metabolic and inflammatory markers assessed. In addition, the current study fills a gap in previous research by examining postprandial lipemia and postprandial inflammation together.

To our knowledge, the present study is among the first to examine the effects of exercise on both postprandial lipemia and inflammation using an exercise duration and intensity that reflect current physical activity guidelines. However, certain considerations should be made when drawing conclusions from the current data. First, partial replacement of the exercise-induced energy deficit (via standard small snack consumption before leaving the laboratory) may have diminished the lipid-lowering effects of the exercise sessions. This post-exercise energy replacement may explain why there was no significant blunting of postprandial lipemia in either exercise trial [35–37]. However, in our opinion consumption of a small snack after the exercise sessions was realistic and reflective of true-to-life behavior. We do acknowledge that the composition of the food item used to replace exercise-induced energy expenditure can have substantial effects on the degree to which the lipid-lowering effects of exercise are negated [51]. Our utilization of a Little Debbie Swiss Roll as a post-exercise snack could have produced effects on postprandial lipemia that may be different from other food items, such as a post-exercise protein shake, and this should be considered when interpreting our results. We assert that our usage of a highly-processed snack as a means of replacing post-exercise energy expenditure coincides with the Western-based test meal, as well as the notion that our studied population (overweight, insufficiently active individuals) may be more likely to consume such a snack compared to a protein shake or something similar. In addition, without a significant effect of exercise on postprandial lipemia, we could not effectively evaluate the association between postprandial changes in triglycerides and inflammation, weakening our ability to support or reject our third hypothesis that there would be a correlation between the postprandial lipemic and systemic inflammatory responses to the high-fat meal. Next, as the test meal was comprised of ice cream and whipping cream, the findings of the present study cannot be extrapolated to other meals with differing macronutrient distribution or nutrient content. Similarly, the test meal was quite large (~1120 kcal), and may not necessarily have been reflective of daily, habitual food intake. Finally, the issue of sample size needs to be addressed. While there were qualitative improvements in the lipemic response during the EX-60 condition compared to CON and EX-30, we did not find any significant statistical differences. It could be suspected that the present investigation was simply under-powered to detect trial differences. However, a *post-hoc* power analysis based on differences between CON and EX-60 (for both AUC-tot and AUC-inc) revealed that an additional 20 participants (32 total) would need to be recruited to the study in order to sufficiently detect trial differences and reject the null hypothesis. The effect size (Cohen’s *d*) for CON vs EX-60 in terms of AUC-tot and AUC-inc lipemic responses were 0.51 and 0.50, respectively, reflecting a moderate effect. Thus, despite a potential lipid-lowering effect of exercise, it is unlikely that the present study was merely underpowered, but rather this issue highlights the ability of post-exercise (partial) caloric replacement to negate the lipid-lowering effects of acute exercise.

## Conclusions

The primary finding of the present study was that when performed 12 h prior to a HFM, neither thirty nor 60 min of aerobic treadmill exercise performed at 60 % VO_2peak_ were adequate to blunt postprandial lipemia in a group of overweight men. Additionally, there were no substantial or noteworthy changes in systemic inflammation between trials. The findings of the present study point to the large exercise dose needed to significantly blunt postprandial lipemia and inflammation following a high-fat meal. On the other hand, the exercise dose may have been sufficient to blunt postprandial lipemia, withholding the partial caloric replacement and near-return to energy balance following exercise. It would be worthwhile for future investigations to continue to determine the volume of realistic exercise (and energy deficit) needed to attenuate postprandial lipemia and systemic inflammation for at-risk individuals in daily life. In addition, consideration of other key parameters, such as body composition, age, and sex of participants, as well as meal size and composition, may provide additional insight into the degree that individuals experience postprandial lipemia in daily life, and the role exercise may play in its attenuation.

## Abbreviations

ACSM, American College of Sports Medicine; AUC-inc, incremental area under the curve; AUC-tot, total area under the curve; CON, control trial (no exercise); DEXA, dual-energy X-ray absorptiometry; EX-30, 30 min of exercise trial; EX-60, 60 min of exercise trial; HFM, high-fat meal; IL, interleukin; LLOQ, lower limit of quality; OGTT, oral glucose tolerance test; REE, resting energy expenditure; RER, respiratory exchange ratio; RMR, resting metabolic rate; TNF, tumor necrosis factor.
